# Surgical Treatment of Haglund’s Deformity: A Systematic Review and Meta-Analysis

**DOI:** 10.7759/cureus.27500

**Published:** 2022-07-31

**Authors:** Wen Loong Paul Yuen, Pei Ting Tan, Kam King Charles Kon

**Affiliations:** 1 Department of Orthopaedic Surgery, Changi General Hospital, Singapore, SGP; 2 Clinical Trials and Research Unit, Changi General Hospital, Singapore, SGP

**Keywords:** posterior calcaneal exostosis, retrocalcaneal bursitis, calcaneal osteotomy, calcaneoplasty, haglund’s deformity

## Abstract

Posterosuperior calcaneal prominence, also known as Haglund’s deformity, can often lead to retrocalcaneal bursitis, a significant cause of posterior heel pain. Surgery is indicated for symptomatic patients, after a period of conservative treatment including analgesia, physiotherapy, activity, and shoe wear modification has failed. Surgical options include both open and endoscopic techniques, and typically involve excision of the retrocalcaneal bursa, resection of the calcaneal prominence, and debridement of the diseased Achilles tendon.

This article aims to provide an evidence-based literature review for the surgical management of Haglund’s deformity. A comprehensive evidence-based literature review of the PubMed database conducted in July 2021 identified 20 relevant articles assessing the efficacy of surgical modalities for Haglund’s deformity. The 20 studies were assigned to a level of evidence (I-IV). Individual studies were reviewed to provide a grade of recommendation (A-C, I) according to the Wright classification in support of or against the surgical modality. Qualitative and quantitative analysis was performed for the 20 studies.

The results show that both open and endoscopic surgical modalities are efficacious in the treatment of Haglund’s deformity, significantly improving functional outcome scores such as American Orthopaedic Foot & Ankle Society (AOFAS) scores and patient satisfaction post-operatively. Endoscopic surgery appears to have the advantage of shorter operative times, lower complication rates, and better cosmesis. More studies are required to further validate and optimize these surgical techniques.

## Introduction and background

Introduction

Haglund’s deformity is an abnormal bony enlargement at the posterosuperior aspect of the calcaneum, first described by Patrick Haglund in 1927 [1]. Repetitive impingement of the retrocalcaneal bursa between the Achilles tendon and the calcaneal prominence can result in retrocalcaneal bursitis, which is a significant cause of posterior heel pain [2,3]. Patients afflicted with this condition typically describe pain localizing to the retrocalcaneal region [4]. Tenderness can be elicited by palpation laterally and medially to the Achilles tendon at the level of the posterosuperior border of the calcaneum, and with ankle dorsiflexion [2]. Classically, posterosuperior calcaneal prominence associated with retrocalcaneal pain and tenderness is called Haglund’s disease.

Lateral X-rays of the ankle can demonstrate posterior calcaneal exostosis, and radiographic measurements such as Fowler’s angle and parallel pitch lines are commonly used to determine the degree of prominence [5]. Ultrasound evaluation of the ankle can be used to evaluate retrocalcaneal bursitis and Achilles tendon degeneration and calcifications. Kondreddi et al. showed that patients with degenerative changes in the Achilles tendon had poor subjective outcomes following endoscopic surgery for Haglund’s deformity [6].

Conservative management is recommended as the first-line treatment for Haglund’s disease. This includes usage of nonsteroidal anti-inflammatory medications, steroid injections, shoe wear modifications (avoidance of tight shoes or those with rigid heel counter, and usage of paddings), physiotherapy (stretching and strengthening gastrocnemius and soleus), and activity modifications [4].

Surgical interventions are typically recommended after a period of conservative treatment has failed, typically six months or more [3]. There is no consensus on the gold standard of surgical treatment for Haglund’s syndrome, and both open and endoscopic techniques have been described, with varying results reported in the literature [7]. Surgical techniques typically involve retrocalcaneal bursectomy, excision of Haglund's deformity, with or without Achilles tendon debridement and reattachment.

For open techniques, the main approaches used are the medial, lateral, and central tendon splitting approach, which typically involves calcaneal resection or wedge osteotomy. Reported complications of open surgical treatment include surgical site infections, altered sensation around the wound and heel, hypertrophic scars, recurrence of pain due to inadequate resection, stiffness, and Achilles tendon rupture [2,8].

Endoscopic techniques potentially offer advantages of minimally invasive surgery including smaller wounds with better scar healing, lower morbidity, shorter recovery time, and quick sports resumption [2,9]. This can be performed utilizing either single or up to three portals, typically involving debridement of the retrocalcaneal bursa and calcaneal resection. Endoscopic surgery however is technically challenging and requires the surgeons to have excellent familiarity with anatomic relationships [10].

The aim of this review was to evaluate the surgical treatment modalities for Haglund’s disease thereby providing a clinical guideline based on the available scientific evidence.

Materials and methods

Sources of Information and Search Strategy

PubMed database was used to perform a thorough literature search, utilizing Preferred Reporting Items for Systematic Reviews and Meta-Analyses (PRISMA) guidelines. The search interval was set for up to 1st June 2021. The search was done using the keywords in English: “Haglund deformity”, “Haglund syndrome”, “retrocalcaneal bursitis”, “endoscopic calcaneoplasty”, “retrocalcaneal decompression”, “calcaneal osteotomy”, “calcaneal ostectomy” and “calcaneal resection”.

Shortlisted papers were reviewed to identify papers with surgical treatments and evaluate their results. Original research reporting clinical and functional outcomes of patients who underwent surgical interventions for Haglund’s deformity with at least 20 patients were considered eligible. Technique papers, review papers without clinical results, case reports, and cadaver studies were excluded. Studies dated prior to January 2000 were also excluded.

**Figure 1:**
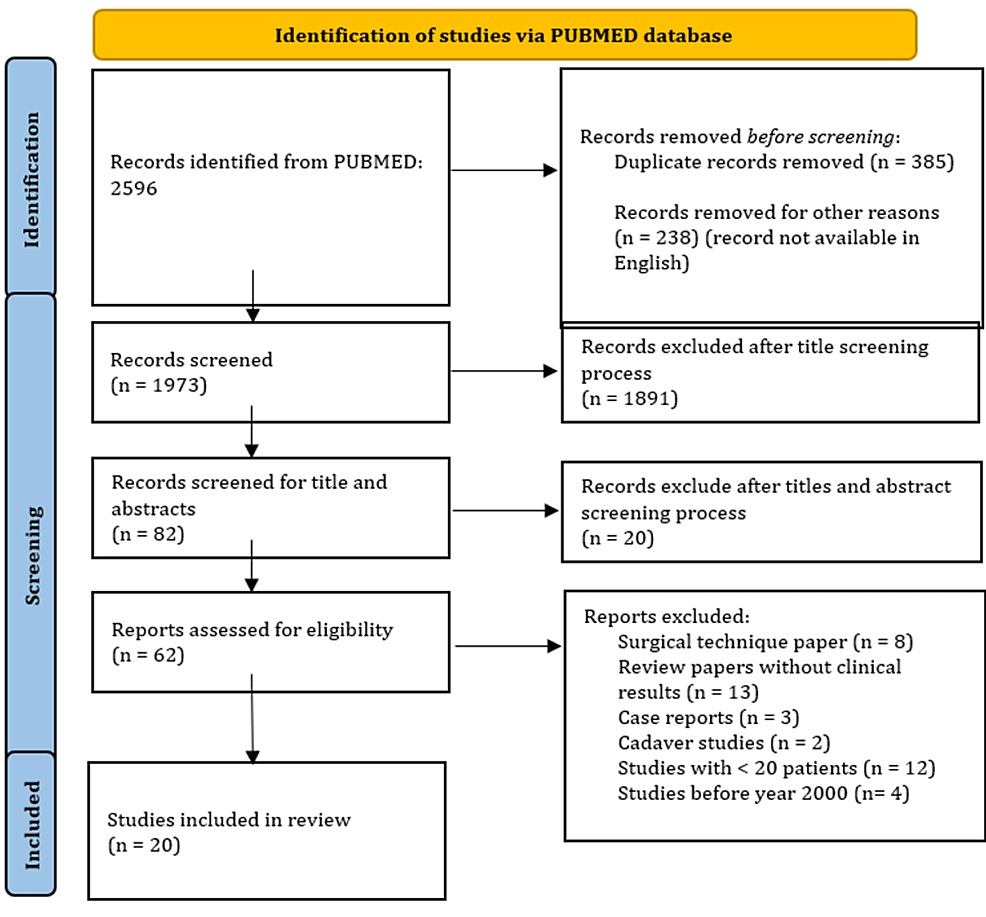
PRISMA flow diagram for surgical modalities PRISMA: Preferred Reporting Items for Systematic Reviews and Meta-Analyses

A total of 2596 studies were identified for initial screening. After duplicate records and records not available in English were removed, 1973 records underwent a title screening process; 1891 studies were excluded as they were not relevant to the topic. The remaining 82 records underwent an abstract screening process, and 20 more were removed due to irrelevance to the topic. Sixty-two records were left and were assessed for eligibility. After exclusion criteria were applied, a final 20 studies were selected to be included in this review (Figure 1).

Each selected study was reviewed and assigned a level of evidence based on the Journal of Bone and Joint Surgery level-of-evidence rating (Table 1) [11]. The studies were reviewed by two orthopedic surgeons and a grade of recommendation (A, B, C, I) was assigned to each intervention based on the classification of Wright (Table 2) [12]. Selected studies with grades of recommendation A to C were analyzed.

**Table 1:** Levels of evidence for surgical modalities

Level	Therapeutic Studies Investigating the Results of Treatment
I	Randomized controlled trial with a significant difference or no significant difference but narrow confidence intervals
II	Prospective cohort study or poor-quality randomized controlled trial (e.g., <80% follow-up)
III	Case-control study or retrospective cohort study
IV	Case series
V	Expert opinion

**Table 2:** Grades of recommendation for orthopaedic surgical studies

Grade	Description
A	Good evidence (Level I studies with consistent findings) for or against recommending intervention
B	Fair evidence (Level II or Level III studies with consistent findings) for or against recommending intervention
C	Poor-quality evidence (Level IV or Level V studies with consistent findings) for or against recommending intervention
I	Insufficient or conflicting evidence not allowing a recommendation for or against intervention

Statistical Analysis

A total of 14 studies that reported pre- and post-operative American Orthopedic Foot & Ankle Society (AOFAS) scores were included for data synthesis. Among these studies, the surgical effect was evaluated for open and endoscopic surgery groups. The measurement of the surgical effect was the change in the AOFAS score, derived from the difference between pre-and post-operative AOFAS scores reported in the studies. The standard deviation (SD) of the change in AOFAS score was derived according to the Cochrane handbook by using the correlation coefficient (r) formula, in which r = 0.74 was applied in this study [13]. Random effects model with the Restricted Maximum Likelihood (REML) method was used to summarize the overall effect of open and endoscopic surgery on the changes in AOFAS with a 95% confidence interval (CI). Heterogeneity between studies was assessed using Cochrane’s Q test and I2 statistics. Sensitivity analysis was performed by including studies with levels of evidence II or III to evaluate the robustness of the result.

Analysis was performed using Stata 16.0 (StataCorp LLC, College Station, TX) and 2-tailed P values <0.05 was considered statistically significant. Qualitative synthesis was also performed to evaluate the surgical interventions reported in the included studies.

## Review

Results

After a PubMed database search, review of articles, and application of exclusion criteria, 20 final studies were selected for analysis. These studies described their surgical treatments for Haglund’s deformity, rehabilitation protocol, and functional outcomes. Table 3 summarizes the general characteristics of selected studies, level of evidence, and grade of recommendation. Table 4 summarizes the studies’ detailed characteristics and surgical outcomes.

**Table 3:** General characteristics of selected studies, level of evidence and grade of recommendation L – Left, R – Right, Y – years, M – months, O – Open, E – Endoscopic; AOFAS - American Orthopaedic Foot and Ankle Society (AOFAS) Ankle-Hindfoot Score; VISA-A - Victorian Institute of Sports Assessment – Achilles Questionnaire; VAS - Visual analogue scale; FFI – foot function index Schneider et al. [14]; van Dijk et al. [8]; Yodlowski et al. [15]; Leitze et al. [4]; Scholten et al. [2]; Jerosch et al. [9]; Ortmann and McBryde [16]; Anderson et al. [17]; Wu et al. [3]; Kondreddi et al. [6]; Kaynak et al. [18]; Natarajan et al. [19]; Jiang et al. [20]; Xia et al. [5]; Yan et al. [21]; Yasin et al. [22]; Ge et al. [23]; Pi et al. [10]; Tourne et al. [7]; Cusumano et al. [24]

S/n	Main Author	Year	Country	Number of Patients	Gender	Number of procedures	Type of Procedure	Side	Mean Age (years)	Follow up range (months)	Scores	Study Design	Level of Evidence	Grade of Recommendation
1	Schneider et al.	2000	Austria	36	13 M 23 F	49	O	L 25 R 24	54.5 (15-70)	4 y 7m (1-11y)	AOFAS	Retrospective case series	IV	C
2	van Dijk et al.	2001	Netherlands	20	10 M 10 F	21	E	L 10 R 11	33.2 (16-50)	3.9 y (2-6.5y)	Ogilve Harris score	Retrospective case series	IV	C
3	Yodlowski et al.	2002	USA	35	25 M 10 F	41	O	(-)	54 (18-83)	39	Pain scale (0-6)	Retrospective cohort study	III	B
4	Leitze et al.	2003	USA	44	14 M 30 F	50	O, E	(-)	50 (15-79)	28 (6-52)	AOFAS, University of Maryland	Prospective cohort study	II	B
5	Scholten et al.	2006	Netherlands	36	20 M 16 F	39	E	L 18 R 21	35 (16-50)	4.5y (2-7.5y)	Ogilve Harris score	Retrospective case series	IV	C
6	Jerosch et al.	2007	Germany	81	40 M 41 F	81	E	(-)	25-55	35.3 (12-72)	Ogilve Harris score	Retrospective case series	IV	C
7	Ortmann and McBryde	2007	USA	30	14 M 16 F	32	E	L 17 R 15	51 (22-75)	35 (3-62)	AOFAS	Retrospective case series	IV	C
8	Anderson et al.	2008	USA	62	22 M 40 F	66	O	(-)	50.5 (19-82)	35 (12-109)	AOFAS, Short form-36	Retrospective controlled study	III	B
9	Wu et al.	2012	China	23	6 M 17 F	25	E	(-)	27.7 (17-41)	41 (30-59)	AOFAS, Ogilve Harris score	Retrospective case series	IV	C
10	Kondreddi et al.	2012	India	23	9 M 14 F	25	E	(-)	51.4 (38-66)	16.4 (12-30)	AOFAS, University of Maryland	Prospective case series	II	B
11	Kaynak et al.	2013	Turkey	28	18 M 10 F	30	E	(-)	37 (19-64)	58.4 (24-75)	AOFAS	Retrospective case series	IV	C
12	Natarajan et al.	2015	India	40	12 M 28 F	46	O	(-)	44 (38-50)	13 (12-15)	AOFAS	Retrospective case series	IV	C
13	Jiang et al.	2016	China	32	11 M 21 F	32	O	L 15 R 17	51.4 (21-68)	42 (24-60)	AOFAS, VISA-A, Arner-Lindholm	Retrospective controlled study	III	B
14	Xia et al.	2017	Singapore	22	10 M 12 F	22	O	L 11 R 11	59.3 (47-77)	15.1 (12-26)	AOFAS, VAS Pain, Short form-36	Retrospective case series	IV	C
15	Yan et al.	2020	China	20	8M 12 F	20	O	(-)	40 (27-57)	32	AOFAS, VAS Pain, Arner-Lindholm	Retrospective case series	IV	C
16	Yasin et al.	2020	Turkey	27	13 M 14 F	27	O	L 13 R 14	47 ± 8 (31-61)	31 ± 5 (21-29)	AOFAS, VAS Pain	Retrospective case series	IV	C
17	Ge et al.	2020	China	44	35 M 9 F	44	O	L 21 R 23	35.5 (18-63)	76 (40-120)	AOFAS, VISA-A	Retrospective case series	IV	C
18	Pi et al.	2021	China	47	36 M 11 F	47	O, E	(-)	37 ± 12	38 ±18	AOFAS, VAS Pain, FFI, Tegner score, ankle activity score, short form-36	Retrospective cohort study	III	B
19	Tourne et al.	2021	France	50	35 M 15 F	50	O	(-)	54 (26-67)	7y (3-10y)	AOFAS, VISA-A, Tegner score	Retrospective cohort study	III	B
20	Cusumano et al.	2021	Italy	54	31 M 23 F	54	O, E	(-)	49 ± 9	53.8 ± 24	AOFAS, VAS, FFI	Retrospective case series	IV	C

**Table 4:** Summary of study characteristics and surgical outcomes Y – years, M – months, D – days; FWB – Full weight bear; PWB – Partial weight bear; WBAT – Weight bearing as tolerated; AT – Achilles tendon; DVT – Deep vein thrombosis; VAS - visual analogue scale; FFI – Foot function index; AAS – Ankle activity score; SF-36 – Short form-36; VISA-A - Victorian institute of sports assessment – Achilles questionnaire; PROMIS – Patient-reported outcome measure information system Schneider et al. [14]; van Dijk et al. [8]; Yodlowski et al. [15]; Leitze et al. [4]; Scholten et al. [2]; Jerosch et al. [9]; Ortmann and McBryde [16]; Anderson et al. [17]; Wu et al. [3]; Kondreddi et al. [6]; Kaynak et al. [18]; Natarajan et al. [19]; Jiang et al. [20]; Xia et al. [5]; Yan et al. [21]; Yasin et al. [22]; Ge et al. [23]; Pi et al. [10]; Tourne et al. [7]; Cusumano et al. [24]

	Main Author	Surgical Intervention			Surgical Details							Results						
		Conservative period before surgery (mean)	Type	Number of procedures	Diagnostic method	Positioning	Anaesthesia	Approach	Details	Time (min)	Post-operative Protocol	AOFAS pre-op	AOFAS post-op	Other Measures	Mean return to work	Mean return to sports	Complications	Revision Surgery
1	Schneider et al.	3y 7m (4w-28y)	Open	49	Xray	Prone	General	Lateral	Calcaneal ostectomy, AT decompression, bursectomy,	-	13 patients FWB post-op, 36 patients PWB with mean of 4w, 10 patients cast for 3-12w	-	93.9 (65-100)	34 complete relief of pain, 7 improvement, 1 no change, 7 worsening	7.5w	20.3w	3 extensive hematoma, 1 superficial infection, 1 recurrent bone spur, 1 foreign body reaction to bone wax, 1 painful ossification	3 revision surgeries for complications (reaction to bone wax, recurrent spur and painful ossification)
2	van Dijk et al.	>6m	Endoscopic	21	Xray	Prone, Supine	General, Regional	2 portals	Bursectomy, calcaneoplasty	48	FWB and ROM post op	-	-	Ogilve Harris: 1 fair 4 good 15 excellent	7w	12w	1 delayed wound healing	Nil
3	Yodlowski et al.	>6m	Open	41	-	Prone	-	Lateral	Bursectomy, AT decompression, calcaneal ostectomy, AT debridement	-	Walker boot with heel lifts and TTWB first 2w, walker boot, ROM and WB after	-	-	Preop pain 4.7 (1.1 SD), post op pain 1.5 (1.3 SD). 14 completely relieved, 17 significantly improved, 4 improved	-	6-9m	14 altered sensations at wound, 1 significant incisions site pain, 1 sural nerve paraesthesia, 1 heel paraesthesia, 1 DVT	Nil
4	Leitze et al.	18m (6-36m)	Open	17	Xray	Supine	General	Medial or lateral	Retrocalcaneal decompression, calcaneal resection	56	Splint and NWB 2w, followed by walker boot with heel lifts and WB	58.1 ± 17.6	79.3 ± 19	3 poor outcomes	-	-	2 superficial infections, 1 delayed wound healing, 2 incisional paraesthesia, 1 heel numbness, 3 incision site tenderness	1 revision surgery (for symptom recurrence)
			Endoscopic	33		Supine	General	1 or 2 portals	Bursectomy, calcaneal resection, AT debridement	44		61.8 ± 12.9	87.5 ± 15	University of Maryland score 86 ± 17, 19 excellent, 5 good, 3 fair, 3 poor	-	-	1 superficial infection, 1 mild sural neuropathy, 1 heel numbness, 1 reflex sympathetic dystrophy-like symptom, 2 port site tenderness	1 revision surgery (for symptom recurrence)
5	Scholten et al.	>6m	Endoscopic	39	Xray	Prone	General or regional	2 portals	Bursectomy, calcaneal resection,	-	WB and ROM post op	-	-	Ogilve Harris: 24 excellent, 6 good, 4 fair, 2 not improved	5w (10d-6m	11w (6w-6m)	1 heel numbness, 1 delayed wound healing	Nil
6	Jerosch et al.	>6m	Endoscopic	81	Xray, MRI	Prone, supine	General or spinal	2 portals	Bursectomy, calcaneal resection	28	PWB 2w	-	-	Ogilve Harris: 41 excellent, 34 good, 3 fair, 3 poor	-	-	1 skin inflammation	3 revision surgery (for symptom recurrence
7	Ortmann and McBryde	-	Endoscopic	32	Xray, MRI	Supine	General or regional	2 portals	Bursectomy, calcaneal resection, AT debridement	-	NWB and splint 2w, followed by PWB with walker boot 2-3w then normal walking	62 ± 12.7	97 ± 6.1	26 excellent, 3 good, 1 poor	-	6-12w	1 Achilles tendon rupture	2 revision surgeries (1 for symptom recurrence, 1 for complication)
8	Anderson et al.	-	Open (lateral)	35	Xray	Prone	-	Lateral	AT debridement, decompression, calcaneal ostectomy	-	Cast 4w followed by walker boot for 4-6w. ROM start 4w, TTWB 6-8w	54 (10-72)	86 (10-100)	Post op SF-36 mental 54 physical 49 1 residual mild-mod pain, 3 severe pains	-	6.5 (4-27)	2 superficial wound infection	Nil
			Open (central)	31		Prone	-	Midline	AT split, decompression, bursectomy, calcaneal ostectomy, AT insertion, repair	-		43 (10-67)	81 (10-100)	Post op SF-36 mental 54 physical 52 1 residual mild-mod pain	-	5.4 (4-21)	2 superficial wound infection, 1 hypertrophic scar, 1 traumatic TA rupture	Nil
9	Wu et al.	>6m	Endoscopic	25	Xray, MRI	Prone	Spinal	3 portals	Bursectomy, calcaneoplasty, AT debridement	34.8 (20-45)	ROM, PWB 2w, FWB after	63.3 ± 11.9	86.8 ± 10.1	Ogilve Harris: 15 excellent, 7 good, 1 fair, 2 poor	-	-	1 wound inflammation	Nil
10	Kondreddi et al.	>6m	Endoscopic	25	Xray, Ultrasound	Prone, Semi-prone	Spinal	2 portals	Bursectomy, calcaneal resection, AT debridement	-	NWB 2w, FWB after	57.9 ± 6.2	89.1 ± 5.3	University of Maryland score 90.3 ± 5.8, 16 excellent, 6 good, 3 fair	-	-	1 wound infection, 2 sural nerve neuropathy, 1 DVT	Nil
11	Kaynak et al.	>6m	Endoscopic	30	Xray, MRI	Prone	General	2 portals	Bursectomy, calcaneal resection,	38 (20-90)	ROM first day, WBAT third day, FWB 2w after	52.6 (24-75)	98.6 (90-100)	-	-	3m	Nil	Nil
12	Natarajan et al.	>6m	Open	46	Xray	-	-	Lateral	Calcaneal ostectomy	-	-	58	86 (60-90)	8 patients with poor outcomes (4 delayed recovery and 4 recurrence of symptoms)	-	-	3 superficial wound infection	Nil
13	Jiang et al.	-	Open (Single row)	16	Xray, MRI	Prone	-	Lateral	AT detachment, debridement, calcaneal resection, AT single row	-	NWB and cast in equinus for 6w, FWB and ROM after	56.1 ± 4.1	81.3 ± 6.5	Preop VISA A score 52.6 ± 5.2, post op 84.1 ± 3.9 Arner-Lindholm 7 excellent 7 good 2 bad 2 symptoms recurrence, 5 mild residual symptoms	-	-	Nil	Nil
			Open (Double row)	16		Prone	-	Lateral	AT detachment, debridement, calcaneal resection, AT double row	-		59.2 ± 6.7	91.1 ± 4.2	Preop VISA A score 50.6 ± 3.2, post op 90.6 ± 3.4 Arner-Lindholm 11 excellent 5 good	-	6m	Nil	Nil
14	Xia et al.	>6m	Open	22	Xray, Ultrasound, MRI	Prone	General	Midline	AT split, partial detachment, debridement, bursectomy, calcaneal resection, AT reattachment	43.4 ± 7.8	Cast with foot in plantigrade 2w, followed by WBAT with walker boot for 4w	39.3 ± 19.5	83 ± 20.7	Preop VAS 7.8 ± 2.0, Post op VAS 1.8 ± 2.7 Pre op SF-36 mental 48.6 physical 36.1 Post op SF-36 Mental 51.1 physical 49.7	-	-	2 delayed wound healing, 1 heel numbness,	-
15	Yan et al.	>3-6m	Open (suture anchor)	10	Xray, MRI	Supine	Spinal	Lateral	Bursectomy, AT debridement, decompression, calcaneal osteotomy, AT insertion with 2 bone anchors	47.1 (38-56)	Cast post op 4w, followed by ankle brace. WB allowed from 6w	47.2	86.3	Pre op VAS 7.6, post op 1.0 Lindholm 7 excellent 3 good	-	-	Nil	Nil
			Open (allogenic graft)	10		Supine	Spinal	Medial	Bursectomy, AT debridement, graft augmentation and AT insertion	59.5 (49-69)		49.4	81.9	Pre op VAS 7.7, post op 1.6 Lindholm 5 excellent 5 good	-	-	Nil	Nil
16	Yasin et al.	>3m	Open	27	Xray	Prone	Regional	Midline	AT central split, bursectomy, AT debridement, calcaneal resection, AT insertion and repair	-	Brace 10 degrees plantar flexion and PWB (50%) 4w, change to brace in neutral, and allow WB next. After 6w remove brace and ROM	47 ± 7	92 ± 4	Pre op VAS 9, post op 2	-	-	3 superficial wound infection	Nil
17	Ge et al.	>6m	Open (dorsal closing wedge osteotomy)	12	Xray	Lateral	Spinal	Lateral	Calcaneal closing wedge osteotomy, calcaneal fixation with 2 partially threaded cannulated screws	-	NWB 4w, passive ROM, PWB with case 6w to 3m, FWB after	52 ± 5.3	98.2 ± 2.3	Preop VISA A score 37.1 ± 5.7, post op 98.2 ± 2.6	-	-	1 delayed union of calcaneus	Nil
			Open (posterosuperior prominence resection)	32		Lateral	Spinal	Lateral	Calcaneal resection	-	NWB 3w, active ROM. PWB after 3w, FWB after 6w	50.7 ± 5.1	93.4 ± 6.1	Preop VISA A score 35.7 ± 7.1, post op 94.3 ± 5	-	-	1 wound infection	Nil
18	Pi et al.	>6m	Open	20	MRI	Prone	-	Lateral	AT Debridement, bursectomy, calcaneoplasty	45 ± 11	Brace post op 2w, change to walker boot, WB and ROM 2-4w, FWB 4-6w	-	96.1 ± 5.0	Post op VAS 0.9 ± 1.2 Post op FFI 2.1 ± 2.7 Post op Tegner score 3.2 ± 1.2 Post op AAS 4.1 ± 1.6 Post op SF-36 Mental 96.8 physical 86.5	8-12w	8-12w	2 transient paraesthesia at surgical site	Nil
			Endoscopic	27		Prone	-	2 portals	Bursectomy, Calcaneoplasty, AT Debridement	65 ± 11		-	92.1 ± 8.0	Post op VAS 1.5 ± 1.8 Post op FFI 3.7 ± 4.7 Post op Tegner score 3.9 ± 1.9 Post op AAS 5.0 ± 2.5 Post op SF-36 Mental 91.3 physical 87.3	8-12w	8-12w	Nil	Nil
19	Tourne et al.	>6m	Open	50	Xray, MRI	-	General and regional	Lateral	Bursectomy, calcaneoplasty, subtraction osteotomy, calcaneal fixation with 6-hole plate, 2 compression, 4 locking screws	-	Cast 2w post op, walker boot after and allow FWB. ROM 6w post op.	50.5 ± 12	88.9 ± 9.9	Preop VISA A score 60.4 ± 10, post op 85.3 ± 15.2 40 excellent, 7 good, 3 fair Tegner scale – 33 returned to sports equivalent to pre-op levels, 13 increased sporting activity, 4 decreased.	-	6w	2 transient paraesthesia, 1 complex regional pain syndrome.	6 patients had removal of metalwork due to pain
20	Cusumano et al.	-	Open	28	Xray	Prone	Spinal	Medial	AT central split, bursectomy, calcaneal resection, AT reattachment and repair	28.3 ± 3.3	Cast 2w post op, followed by splint and ROM. NWB 1m total	65.7 ± 10.1	91.8 ± 9.7	Preop VAS 6.3 ± 1.4, Post op VAS 1.2 ± 1.9 FFI (Pain) Pre – 52.2 ± 13.3 Post – 8.4 ± 17.1 FFI (Disability) Pre - 53.4 ± 11.7 Post – 9.3 ± 17.4 2 recurrences of symptoms	-	6-12w	1 AT rupture	2 revision surgery (for symptom recurrence
			Endoscopic	26		Prone	Regional	2 portals	Bursectomy, calcaneoplasty	30 ± 4.7	Progressive WB day after surgery	66.7 ± 7.2	93.7 ± 9.7	Preop VAS 7.6 ± 1.3, Post op VAS 1.3 ± 1.9 FFI (Pain) Pre - 55.9 ± 12.3 post - 9.6 ± 16.5 FFI (Disability) Pre – 48.7 ± 12.9 Post – 7.3 ± 12.8 1 recurrences of symptoms	-	6-12w	2 wound infections	1 revision surgery (for symptom recurrence 1 revision surgery for complications (wound revision after infection)

Conservative management

All studies trialed conservative management before surgical intervention. Majority of the studies had a period of conservative management for at least six months before performing surgery [2-10,15,18,19,23]. Two of the included studies intervened surgically after three months of conservative treatment [21,22].

Surgical techniques

The studies have described several surgical techniques for the treatment of Haglund’s deformity. The main comparisons were drawn for open surgery versus endoscopic interventions. For open surgeries, three main approaches were used. They were the lateral approach [4,7,10,14,15,17,19-21,23], medial approach [4,21,24], and midline Achilles tendon central split approach [5,17,22]. There were two studies that utilized the dorsal closing wedge osteotomy technique [7,23]. For open surgery, majority of the studies positioned their patients prone for surgery [5,10,14,15,17,20,22,24], while two studies positioned their patients supine [4,21], and one study positioned their patients lateral [23].

For endoscopic surgeries, single [4], two [2,4,6,8-10,16,18,24] and three portal techniques [3] have been described. Majority of the studies positioned their patients prone for endoscopic surgery [2,3,6,8-10,18,24], while four studies positioned their patients supine [4,8,9,16]. The operating time for endoscopic surgery averaged 38.2 min (range 28-65 min), significantly shorter (p < 0.001) compared the operating time for open surgery, which averaged 43.6 min (range 28 -56 min).

Outcome measures

Different measures were used to evaluate surgical outcomes. The most commonly used measure was the AOFAS score [3-7,10,14,16-24]. Five papers utilized the visual analogue scale (VAS) score [5,10,21,22,24] and four papers utilized the Ogilve-Harris score [2,3,8,9]. Three papers utilized the Victorian Institute of Sports Assessment - Achilles questionnaire (VISA-A) [7,20,23] and Short form-36 (SF-36) [5,10,17] each. Two papers utilized the Foot function index (FFI) score [10,24], TEGENER scale [7,10], Arner-Lindholm scale [20,21] and University of Maryland score [4,6] each. One paper utilized the Ankle activity score (AAS) [10] and Pain scale (score 0-6) [15] each.

Complications

The complication rate for open surgery was 12.3% (ranged from 0-53%). The complications reported included transient paresthesia around the wound (4.3%), superficial infection (3%), incision site tenderness (0.8%), delayed wound healing (0.6%), heel numbness (0.6%), extensive hematoma (0.6%), Achille’s tendon rupture (0.4%), complex regional pain syndrome (0.2%), hypertrophic scar (0.2%), sural nerve neuropathy (0.2%) and deep vein thrombosis (0.2%)

The rates of repeat or revision surgery was 2.6% (ranged from 0-12%). Several cases of re-operation (removal of metal implants) were noted for closing wedge osteotomy group due to pain from the metalwork [7]. There was also one reported case of delayed calcaneal union from closing wedge osteotomy [23].

The complication rate for endoscopic surgery was 5.3% (ranged from 0-18%). The complications reported included superficial wound infection/inflammation (1.8%), sural nerve neuropathy (0.9%), delayed wound healing (0.6%), heel numbness (0.6%), port site tenderness (0.6%), deep vein thrombosis (0.3%), Achilles tendon rupture (0.3%) and complex regional pain syndrome (0.3%). The rates of repeat or revision surgery was 2.4% (ranged 0-7.7%)

Rehabilitation and return to work/sports

Patients in the open surgery studies were often immobilized with cast or brace post operatively, for a period of two weeks up to six weeks [4,5,7,10,17,20-22,24]. Several studies then placed the patients with either an articulated splint [24] or walker boot [4,5,7,10,17,21] after the initial period of casting. Patients were kept non-weight bearing post-op ranging from period of zero up to six weeks [4,5,7,10,15,17,20-24]. Return to normal daily activities and sports ranged from six to 36 weeks [7,14,15,17,19,20,24].

Patients in the endoscopic surgery were immobilized with cast or brace for up to 2 weeks for several studies [4,10,16]. Several studies used a walker boot after the initial period of casting [4,10,16]. Patients were kept non-weight bearing post-op ranging from period of zero up to two weeks [2-4,6,8-10,16,18,24]. Return to normal daily activities and sports ranged from six to 12 weeks [2,8,16,18,24]

Grade of recommendation

The grades of recommendation assigned for each intervention is summarized in Table 5.

**Table 5:** Summary of grade of recommendation for surgical modalities for Haglund’s deformity

Surgical Intervention	Number of Studies	Level I	Level II	Level III	Level IV	Grade	Recommendation
Open Surgery (study for intervention)	12	-	1	5	6	B	Fair evidence for recommending intervention
Open Surgery (study against intervention)	1	-	-	-	1	C	Poor-quality evidence for recommending against intervention
Endoscopic Surgery	10	-	2	1	7	C	Poor-quality evidence for recommending intervention

Studies with both open and endoscopic surgery

There were three head-to-head studies comparing open and endoscopic surgery [4,10,24]. Leitze et al. performed a prospective cohort study comparing endoscopic retrocalcaneal decompression against open technique (50 heels in 44 patients) over 22-months follow-up (level II). They found that the endoscopic group had significant improvements in AOFAS scores from 61.8 preoperatively to 87.5 post operatively, while the open group also had significant improvements in AOFAS scores from 58.1 to 79.3. They found that post-surgical endoscopic scores were numerically but not significant better then open scores, and that endoscopic surgery had shorter surgical times and fewer complications. They concluded that endoscopic surgery is feasible and efficient, and produces results equal or better to open surgery. Potential limitations of the study was the small sample size for the open group, which was also not prospectively recruited [4].

Pi et al. conducted a retrospective cohort study comparing open versus endoscopic calcaneal osteotomy for 47 patients (level III). They found that there were no significant differences between outcome scores of both groups. They concluded that endoscopic surgery was as effective as open procedure, however, it required significantly more surgical time. They also found that the duration of endoscopic surgery shortened after fourth surgery, suggesting that there was a learning curve, requiring sound technical skills and familiarity with anatomic relationships. The authors felt the study had limitations due to the lack of randomization, included results from the learning curve for endoscopic procedures, did not include pre-operative outcome measures and that the study group was of low demand patients [10]. 

Cusumano et al. performed a retrospective review of 54 patients who underwent transtendinous open calcaneoplasty versus endoscopic calcaneoplasty (level IV). Both groups had significant improvements in AOFAS with open group improving from 65.7 to 91.8 and endoscopic group improving from 66.7 to 93.6. They found no significant difference between outcome scores with similar satisfaction rates. They concluded that both techniques provide good clinical outcomes with low rates of complications. The study did not have randomization, and had different post-operative immobilization protocols for the two groups [24].

Studies for Open Surgery

The level of evidence supporting open surgery ranged from level II to level IV. The highest quality study examining the outcome of open surgery was a level II study [4]. One level IV study advised caution against recommending open surgery [14].

Yodlowski et al. conducted a retrospective cohort study on a group for patients (41 heels in 35 patients) who also underwent open retrocalcaneal bursectomy and partial calcaneal exostectomy via lateral approach over 20-months follow-up (level III). They also performed an Achilles tendon intrasubstance debridement if there were calcifications noted intraoperatively. They found that patients postoperative had significant reduction in pain score from 4.7 to 1.5 (out of 6), with 14 patients describing complete relieve of symptoms, 17 significantly improved and 4 improved. They concluded that open surgical treatment is an effective treatment. The study did not utilize AOFAS scores for comparison [15].

Anderson et al. performed a retrospective comparative study on a group of patients (66 feet in 62 patients) and compared the outcomes between open surgery via lateral approach against midline tendon-splitting approach (level III). They found that the AOFAS score improved significantly for both groups, 43 to 81 for tendon-splitting group and 54 to 86 for lateral approach group. They concluded that both approaches provided symptomatic pain relief, but noted that the tendon-splitting group returned to normal functions quicker. Limitations of study include lack of randomization and short-follow up for tendon-splitting group [17].

Jiang et al. conducted a retrospective controlled study on a group of 32 patients and compared the outcomes of open surgery with reattachment of Achilles tendon via double row versus single row after detachment at its insertion and debridement as part of treatment for Haglund’s syndrome over 3.5 years follow-up (level III). Both groups had significant improvements in AOFAS scores with single row group improving from 56.1 to 81.3 and double row group improving from 59.2 to 91.1. They concluded that double row suture technique appeared to be a better option with favorable surgical outcomes. There was lack of randomization between the groups [20].

Natarajan et al., Xia et al., Yasin et al., and Yan et al. all performed retrospective case series respectively which showed good outcomes for open surgery (level IV). [5,19,21,22]. Natarajan et al. conducted a retrospective study on a group of patients (46 feet in 40 patients) who underwent open calcaneal resection via lateral approach over a period of one year follow-up (level IV). Their results showed improvement of 28 points to 86 for AOFAS scores with majority of patients reporting alleviation of pain at one year follow-up. They concluded that open surgery is an effective treatment, however recovery period to obtain maximum benefit is longer (six months) [19].

Xia et al. performed a retrospective study of 22 patients who underwent open calcaneal resection via midline splitting approach over 15 months follow-up (level IV). Their results showed significant improvements in AOFAS scores from 39.3 to 83.0. They concluded that open surgery via midline splitting approach is effective, providing pain relief, functional improvement and overall enhancement of patient’s health. Limitations for the study include small sample size and short follow-up [5].

Yasin et al. conducted a retrospective study of 27 patients who underwent open calcaneal resection and Achilles tendon debridement via midline tendon splitting approach and double row suture anchor repair over 30 months follow-up (level IV). Their results showed significant improvements in AOFAS from 47 to 92. They concluded that their technique was effective and safe treatment option. Limitations for the study include lack of control group and small sample size [22].

Yan et al. retrospectively studied 20 patients who underwent open calcaneal osteotomy with Achilles tendon debridement with repair with bone anchor versus allogenic tendon augmentation (level IV). They found both groups had significant improvement in AOFAS, with bone anchor group improving 47.2 to 86.3 and allogenic tendon group 49.4 to 81.9. They found that suture anchor group was more suitable for open Haglund’s surgery. Limitations for the study include small sample size and short follow-up [21].

Two studies described dorsal closing wedge osteotomy [7,23]. Tourne et al. performed a retrospective cohort study of 40 patients who underwent closing wedge osteotomy over 7 years follow-up (level III) [7]. They found significant improvements in AOFAS scores from 50.5 to 88.9 with 40 excellent, seven good and three fair results. They concluded that closing wedge osteotomy is an efficient and reliable way to change the configuration of the Achilles tendon insertional area and support the efficacy of calcaneoplasty [7]. Ge et al. conducted a retrospective study of 44 patients comparing the outcomes of dorsal closing wedge osteotomy versus posterosuperior prominence resection (level IV). Both groups of patients had significant improvements with AOFAS score improving from 52 to 98 for the dorsal closing wedge osteotomy group and 51 to 93 for the posterosuperior prominence resection group. They found that the dorsal closing wedge osteotomy group had poorer short term outcomes but better functional improvement during long term follow-up [23].

One study recommended against open surgery. Schneider et al. conducted a retrospective study on a group of patients (49 heels in 36 patients) who underwent open calcaneal resection via lateral approach over a four-year follow-up (level IV). They reported seven complications, three requiring revision surgery, and seven patients who reported worsening of their symptoms post-operatively, with only 73.5% satisfaction amongst operated patients. They concluded that open surgical treatment often results in unsatisfied patients, advised caution for recommending treatment and that all possible and prolong conservative treatments should be completed first. The study did not compare pre-operative AOFAS to post-operative scores [14].

Grade of recommendation: Based on the previously mentioned literature, open surgical treatment of Haglund’s deformity is assigned a grade B recommendation (fair evidence, level II or III studies with consistent findings).

Studies for Endoscopic Surgery

The level of evidence supporting endoscopic surgery ranged from level II to level IV. The highest quality study examining the outcome of endoscopic surgery was a level II study [6]. Kondreddi et al. conducted a prospective study on a group of patients (25 heels in 23 patients) who underwent endoscopic retrocalcaneal decompression, calcaneal resection, and debridement of unhealthy Achilles tendon over one year follow-up (level II). The patients had significant improvement in preoperative AOFAS from 57.92 to postoperative score of 89.08. They concluded that endoscopic calcaneal resection was highly effective for patients with mild or no degenerative changes in Achilles tendon, had better cosmetic outcomes and fewer complications. Patients with degenerative changes in Achilles tendon had poorer outcomes in terms of subjective satisfaction. Limitations for the study include small sample size and short follow-up [6].

Van Dijk et al., Scholten et al., Jerosch et al., Ortmann and McBryde, Wu et al., and Kaynak et al. all performed retrospective case series respectively which showed favorable outcomes for endoscopic surgery (level IV) [2,3,8,9,16,18]. Van Dijk et al. conducted a retrospective study on a group of patients (21 heels in 20 patients) who underwent endoscopic calcaneoplasty over four years of follow-up (level IV). They found all but one patient had improved symptoms, with 15 excellent, four good and one fair result, and no complications. They found that endoscopic surgery has low morbidity, patients have shorter recovery, and patients quickly resume work and sports. The study population was small and did not utilize AOFAS score for comparison [8].

Scholten et al. performed a retrospective study for a group of patients (39 heels in 36 patients) who also underwent endoscopic calcaneoplasty over 4.5 years of follow-up (level IV). Their results showed 24 excellent results, six good results, four fair results, and only two were not improved. They concluded that endoscopic surgery had good results, and had several advantages including low morbidity, excellent scar healing, short recovery time, and quick sports resumption. The study did not utilize the AOFAS score for comparison [2].

Jerosch et al. conducted a retrospective study on a group of 81 patients who underwent endoscopic calcaneoplasty over a period of 35 months follow-up (level IV). Their results showed 41 excellent, 34 good, three fair, and only three poor results. They concluded that endoscopic surgery is effective with a short learning curve, and appeared to less morbidity, less operating time and nearly no complications. The study did not utilize the AOFAS score for comparison [9].

Ortmann and McBryde performed a retrospective study on a group of patients (30 heels in 28 patients) who underwent endoscopic bony and soft tissue decompression of retrocalcaneal space over a period of 35 months follow-up (level IV). Their results showed significant improvement in AOFAS scores from 62 to 97, with 26 excellent, three good, and only one poor result. They concluded that endoscopic surgery has low morbidity, high patient satisfaction, with short return to normal activity. There were limitations of the study including the small cohort and retrospective collection of AOFAS scores for some patients [16].

Wu et al. conducted a retrospective study for a group of patients (25 heels in 23 patients) who underwent endoscopic calcaneoplasty via a three portal (proximal posterolateral, distal posteromedial portal and distal posterolateral portals) approach over a period of 41 months (level IV). They found that their patients had significant improvements in AOFAS from 63.3 to 86.8 with 14 excellent, seven good, one fair and only two poorly results without any complications. They concluded that endoscopic surgery was safe and efficacious. The authors felt the study was limited due to small patient population, short follow-up duration and lack of control group [3].

Kaynak et al. performed a retrospective study for a group of patients (30 heels in 28 patients) who underwent endoscopic calcaneoplasty over 58 months (level IV). Their results showed improvements in AOFAS scores from 52.6 to 98.6 without any complications. They concluded that endoscopic surgery was a safe and effective treatment option [18].

Grade of recommendation: Based on the previously mentioned literature, endoscopic surgical treatment of Haglund’s deformity is assigned a grade C recommendation (poor evidence, level IV or V studies with consistent findings)

Meta-analysis

**Figure 2:**
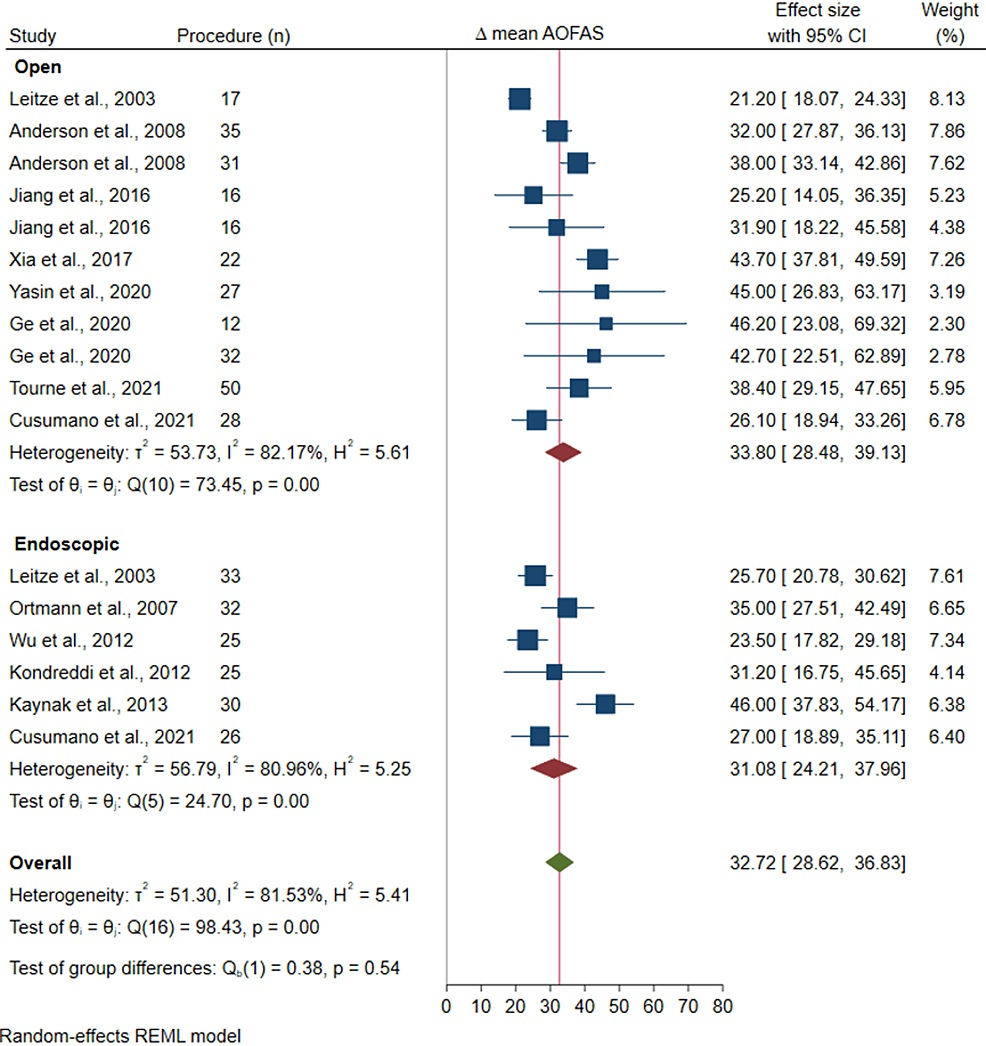
Forest plot for change in AOFAS scores for open and endoscopic surgical subgroups American Orthopaedic Foot & Ankle Society Leitze et al. 2003 [4]; Anderson et al. 2008 [17]; Jiang et al. 2016 [20]; Xia et al. 2017 [5]; Yasin et al. 2020 [22]; Ge et al. 2020 [23]; Tourne et al. 2021 [7]; Cusumano et al. 2021 [24]; Ortmann and McBryde 2007 [16]; Wu et al. 2012 [3]; Kondreddi et al. 2012 [6]; Kaynak et al. 2013 [18]

The results showed that treatment with both open and endoscopic surgery lead to statistically significant improvements in AOFAS scores; 33.8 (95% CI: 24.5 to 39.1, p < 0.001) and 31.1 (95% CI: 24.2 to 38.0, p < 0 .001) respectively (Figure 2). There was no significant difference between the two surgical groups (mean difference in AOFAS change score between open and endoscopic surgery was 2.79 (95%CI: -0.73 to 6.32; I2=87.9, P-heterogeneity <0.001). Sensitivity analysis results also showed a similar result with no significant difference between open and endoscopic surgical groups (31.0 95%CI: 24.8, 37.1 vs 26.3 95%CI: 25.2, 35.1). 

Discussion

Haglund’s deformity remains a significant cause of posterior heel pain. Surgery is indicated after a period of conservative management has failed, with most authors preferring at least six months of conservative treatments. Principles of surgery include excision of inflamed retrocalcaneal bursa, resection of Haglund’s deformity, and debridement of unhealthy Achilles tendon [8]. The gold-standard approach to this procedure has not been determined. Our study reviewed the evidence for surgical treatment of Haglund’s deformity over the past 20 years.

Both open and endoscopic surgical techniques have shown promising results in improving AOFAS scores and patient satisfaction. Open surgery can be done via medial, lateral or midline tendon-splitting approaches. Dorsal closing wedge osteotomy is an option for open surgery. Endoscopic surgery can be performed via one to three portal approaches. Both techniques have similarly shown to improve AOFAS scores significantly, 33.8 for open group and 31.1 for endoscopic group. There was no significant difference in improvement of outcome scores for the two groups.

The complication rates for open surgery group were about 12.3% while endoscopic group were lower at 5.3%. The type of complications amongst the groups were similar. The two groups also had similar rates of revision or repeat surgery (2.6% in open vs 2.4% in endoscopic). The most common complications reported by the open surgery group were wound-related complications (including superficial infections, transient paresthesia, incisional site tenderness, delayed wound healing, hypertrophic scar), heel numbness and extensive hematoma formation. The most common complications reported by the endoscopic surgery group were similarly wound-related complications (superficial infections, delayed wound healing, port site tenderness), heel numbness and sural nerve neuropathy. Both groups reported low rates of major complications, including deep vein thrombosis (one in each group) and Achilles tendon rupture (two in open, one in endoscopic group).

Endoscopic surgery offers an excellent alternative to open surgery. The use of the arthroscope potentially allows for better visualization [8]. One of the cornerstones for successful surgery is adequate calcaneal resection, which requires good exposure [3]. This potentially requires a larger incision in open surgery which can result in significant wound and soft tissue complications [8]. This may explain some of the differences in complication rates, favoring endoscopic surgery.

Achilles tendon rupture is a potential major complication following surgical treatments. Excessive calcaneal resection and Achilles tendon debridement are risks factors [24]. Based on a biomechanical study by Kolodziej et al., up to 50% of the tendon can be resected from superiorly to inferiorly safely [25]. Ortman and McBryde reported one case of Achilles tendon rupture three weeks following endoscopic calcaneoplasty, which required primary repair [16]. Anderson et al. reported a case of traumatic Achilles tendon rupture in patient who fell three months after open calcaneoplasty [17]. Cusumano et al. reported a case of Achilles tendon rupture 37 days post open surgery treated conservatively with casting for six weeks. Ortman and McBryde suggested that there may be difficulty with judging depth of Achilles tendon debridement endoscopically, and open surgery may be favored for this instance. He also suggested that longer period of cast immobilization for patients who have also underwent Achilles tendon debridement. However, it is also noted that endoscopic surgery potentially allows better visualization of the calcaneal prominence, and allows more precise local decompression and avoid unnecessary bony over-resection [26].

Operating time and steep learning curve for endoscopic calcaneoplasty have been sources of concern. Jerosch found that after their first 10 cases (mean operating time 46 min), their mean operation time was much reduced to 25 min [9]. Similarly, Leitze found that there was a steep learning curve for the endoscopic procedure, with their first case taking up to two hours which greatly improved to an average of 30 min toward the end of their study [4]. Kaynak found similar experiences in their study, with their operating times improving from 90 min for initial cases to averaging 20-30 min [18]. Pi et al. highlighted the importance of high technical knowledge with good surgical skills and understanding of anatomic relationships for successful endoscopic surgeries [10]. Our study showed that on average, operating time for endoscopic surgery was significantly shorter than open surgery.

Wu et al. suggest further optimization of endoscopic surgery with their three-portal technique. They felt that two portal techniques had difficulty with acquiring convenient manipulation simultaneously with adequate viewing of the calcaneal prominence, and with the limited working space between the two portals place an inherent risk of damage to damaging structures or instruments. They utilize an additional portal, the proximal posterolateral portal mainly as a viewing portal [3].

There are several limitations to our study. There is a significant lack of high-quality evidence, with only two prospective studies. The other studies were level III (five studies) and level IV (13 studies). Majority of the studies were retrospective in nature which are subjected to significant biases. Minor complications may not have been reported which would affect overall complication rates. Due to small number of studies available, results for different approaches (medial, lateral, midline tendon splitting, closing wedge osteotomy) were pooled together for the open surgical group. Similarly, results for different approaches (one to three portals) for endoscopic groups were also pooled together for comparison. It is also important to note there was substantial heterogeneity among the study populations. Our study is also subject to publication bias as they may be other negative studies which are not published.

Our study shows that surgical treatment of Haglund’s deformity results in good clinical outcomes. Endoscopic surgery for Haglund’s deformity is a safe and efficacious treatment approach with lower complication rates and shorter surgical times. Due to lack of high-quality studies, it only has a grade C recommendation. More high-level evidence research such as randomized controlled trials or prospective studies should be done to further validate and optimize this technique.

## Conclusions

Both open and endoscopic surgery for Haglund’s deformity has shown to achieve good results, significantly improving functional outcome scores such as AOFAS scores and patient satisfaction post operatively. Endoscopic surgery for Haglund’s deformity is non-inferior to open surgery. Potential benefits for endoscopic surgery include better cosmesis, shorter surgical times and lower complication rates. There is a steep learning curve for endoscopic surgery, and requires good surgical skills and understanding of anatomic relationships. More studies are required to further validate and optimize these surgical techniques.
